# Frequency of CMV testing during pregnancy-a retrospective study

**DOI:** 10.1007/s00404-025-07962-3

**Published:** 2025-01-31

**Authors:** A. Hadjiiona, I. Michaelides, P. Kummer, M. Kappelmeyer, A. Koeninger, E. Reuschel

**Affiliations:** 1 University Department of Obstetrics and Gynecology, Clinic St. Hedwig of The Order of St. John, University of Regensburg, Steinmetzstrasse 1-3, 93049 Regensburg, Germany; 2 Department of Otorhinolaryngology, Head and Neck Surgery, University Hospital Regensburg, Regensburg, Germany; 3 Department of Otorhinolaryngology, Department of Phoniatrics and Pediatric Audiology, University Hospital Regensburg, Regensburg, Germany; 4 Chair of Obstetrics and Gynecology, Focus: Obstetrics, University of Regensburg, Biopark 1, Regensburg, Germany

**Keywords:** Cytomegalovirus, Prenatal care, Congenital infection, Pregnancy

## Abstract

**Purpose:**

The cytomegalovirus (CMV) belongs to the family of human Herpesviridae and is distributed worldwide. It is the most common cause of viral congenital infections and can have serious consequences for the health of the fetus in the event of a vertical transmission. This study, taking place for the first time in Upper Palatinate, Bavaria, aims to evaluate the frequency of CMV testing among pregnant women in our region in Germany, which for some individuals can be an expensive individual health service.

**Methods:**

Retrospectively, 1000 pregnant patients aged 17–45 years who were treated in the University Clinic St. Hedwig, Regensburg, Germany, were included in the study. It was investigated whether a CMV test was carried out during pregnancy and which results were obtained.

**Results:**

597 patients (59.7%) had not received a CMV test during pregnancy. Among the 403 (40.3%) patients who had undergone CMV testing, seropositivity was detected in 143 (35.5%). 257 patients (63.8%) were seronegative, while 3 (0.74%) had a primary infection.

**Conclusion:**

Although CMV is the most common pathogen of viral congenital infections and can severely impair the health of affected newborns, CMV diagnostics during pregnancy is still not an integral part of the maternity guidelines in Germany, but rather an individual healthcare service, meaning that the patients undergoing the test must bear the full cost. An antiviral treatment with valacyclovir has shown good preventive and therapeutic success, but unfortunately, there is currently no vaccination available to prevent vertical transmission, which is why early diagnosis and hygiene measures are the most important means of preventing seroconversion of the mother and possible infection of the fetus.

**Supplementary Information:**

The online version contains supplementary material available at 10.1007/s00404-025-07962-3.

## What does this study add to the clinical work

**Table:** 

Even though CMV can severely impact the health of newborns, CMV testing is not offered as part of the standard prenatal care in Germany and its’ costs are not covered by the health insurance. Our study, the first one taking place in Upper Palatinate, Bavaria, concerning this subject, showed that the majority of the pregnant women in Germany do not undergo a CMV testing and most of those tested, are seronegative.	

## Introduction

Human Cytomegalovirus belongs to the family of herpes viruses and is the most common pathogen of viral congenital infection [1]. The prevalence depends on various factors e.g. socioeconomic status, hygiene, age, number of sexual partners, contact to toddlers [2]. Immunocompetent adults are usually asymptomatic in case of a CMV infection, whereas it can be dangerous for immunocompromised individuals [3].

CMV can be transmitted through contact with infected body fluids [4]. A vertical transmission can cause a congenital infection. A maternal primary infection can lead to severe outcomes concerning fetus’ health [5]. The risk of transmission increases with gestational age, whereas the risk of fetal impairment decreases [6, 7]. The severity of the symptoms in infected newborns is diverse including hepatosplenomegaly, thrombocytopenia, hemolytic anemia, microcephaly, chorioretinitis, intrauterine growth restriction, low birth weight, motor and cognitive disorders, visual impairment as well as sensorineural hearing loss (SNHL) [8]. The majority of infected newborns are asymptomatic at birth, yet some can develop late-onset-SNHL [9].

Early intervention in case of cCMV is crucial for the outcome, therefore early diagnosis during pregnancy can have a positive impact on the affected newborns [10]. Most pregnant women are not sufficiently informed about cCMV and the relevance of preventive measures [11], thus educating pregnant women about CMV and adhering to hygiene measures are currently the most important measures for preventing seroconversion during pregnancy [12]. Momentarily, there is no validated vaccine during pregnancy, that can prevent the vertical transmission but valacyclovir showed a significant reduction of transmission in several randomized trials [13–16] Unfortunately, in Germany CMV testing in the first trimester is offered as part of an individual healthcare service i.e. the test is carried out upon patient request or after recommendation and the patients have to cover the full costs themselves.

Aim of our study is to evaluate for the first time in Upper Palatinate, Bavaria the frequency of CMV testing during pregnancy as an individual healthcare service, since in Germany the available literature to the subject is very limited.

## Materials and methods

This retrospective, anonymized single center cohort study was performed from June 2021 until August 2022 at the Tertiary Maternity Clinic St. Hedwig of The Order of St. John, University Department of Obstetrics and Gynecology, University of Regensburg. Data of 1000 pregnant women, who were treated and delivered in our clinic, were collected from their medical records. The data analyzed, included maternal date of birth, age, gravidity- and parity-status, CMV serostatus (IgG positive/ IgM negative—seropositive patients, IgG negative/ IgM negative—seronegative patients and IgG positive/ IgM positive and low IgG avidity—patients with a primary infection), due date, date of delivery, gestational age at delivery and sex of the newborn. In cases of primary infection, details about therapy and neonatal outcome were registered and reported.

Data collection was performed using SAP® (© 2024 SAP SE), Viewpoint 6 (Generic Electric Company, Boston, USA) and the “maternity health passport”, a documentation booklet that is part of maternal care following the maternity guidelines in Germany and saved anonymously in Microsoft Excel® for MacOS (Microsoft Corporation, 2022, Version 16.67), therefore an informed consent of the patients was not required. The statistical analysis was performed using GraphPad PRISM 10.2.3® (MacOS, GraphPad Software, San Diego, California, USA).

The CMV testing was performed at 10–12 weeks of gestation at the private practices of the patients’ gynecologists, where the prenatal care check ups take place in Germany, in the form of chemiluminescent magnetic microparticle immunoassay (CMIA) using serum samples of the patients, testing for CMV IgG and IgM antibodies. The results of CMV testing for our study were obtained from the “maternity health passports”, which the patients presented upon admission to our clinic.

Quantitative real-time polymerase chain reaction (qPCR) for the detection of CMV DNA from plasma, serum and in one case from amniotic fluid was performed in patients with a primary infection with an in-house assay at the institute of clinical microbiology (University Medical Center Regensburg, Germany). Nucleic acid was extracted on an EZ1 Advanced XL workstation using the EZ1 Virus Mini Kit v2.0 (Qiagen) with 100 μL elution. A 5‐μL aliquot of the eluate was used for qPCR with the TaqMan 2 × Universal PCR Master Mix (Applied Biosystems). Two replicates were analyzed in 30 μL reactions including ROX buffer, an internal control (Volvox carteri plasmid), MgCl2, dNTPs, specific primers (300 nmol/L each), and TaqMan hydrolysis probe (100 nmol/L). Primers and probes (Cap1-fw, 5'-cag cct acc cgt acc ttt cca-3'; Cap2-rev, 5'-gcg ttt aat gtc gtc gct caa-3'; and Cap-so, FAM-5'-ttc tac tca aac ccc acc atc tgc gc-3'-TAMRA) for the CMV RT‐qPCR are located in major capsid protein (ccapA) gene of the CMV genome. Thermal cycling was done on a StepOnePlus instrument (Applied Biosystems) and comprised an initial step at 95 °C for 10 min, followed by 45 cycles of 95 °C for 15 s and 60 °C for 60 s. The assay was calibrated against the WHO International Standard (NIBSC code number 09/162) and CMV DNA was quantified as copies/mL and International Units per mL (IU/mL), where 0.60 copies equal 1.0 IU. The lower limit of detection is 300 copies/mL (498 IU/mL).

A t-test was used to compare the means of normally distributed continuous values. The Mann–Whitney U-test was used to compare two non-normally distributed continuous groups. For contingency tables, Fisher's exact test was used. One-way Welch’s ANOVA was used to compare the means of more than two normally distributed groups with unequal standard deviations assumed. For post-hoc multiple comparisons Dunnett’s T3-test was used. A p-value of < 0.05 was considered statistically significant.

## Results

### Patient characteristics

1000 pregnant women, aged between 17 to 45 (mean: 32.29 years, SD = 4.76), were enrolled in the study. All newborns were live births (488 females 535 males, 977 singletons, 23 twin births).

### Frequency of CMV testing

**Fig. 1:**
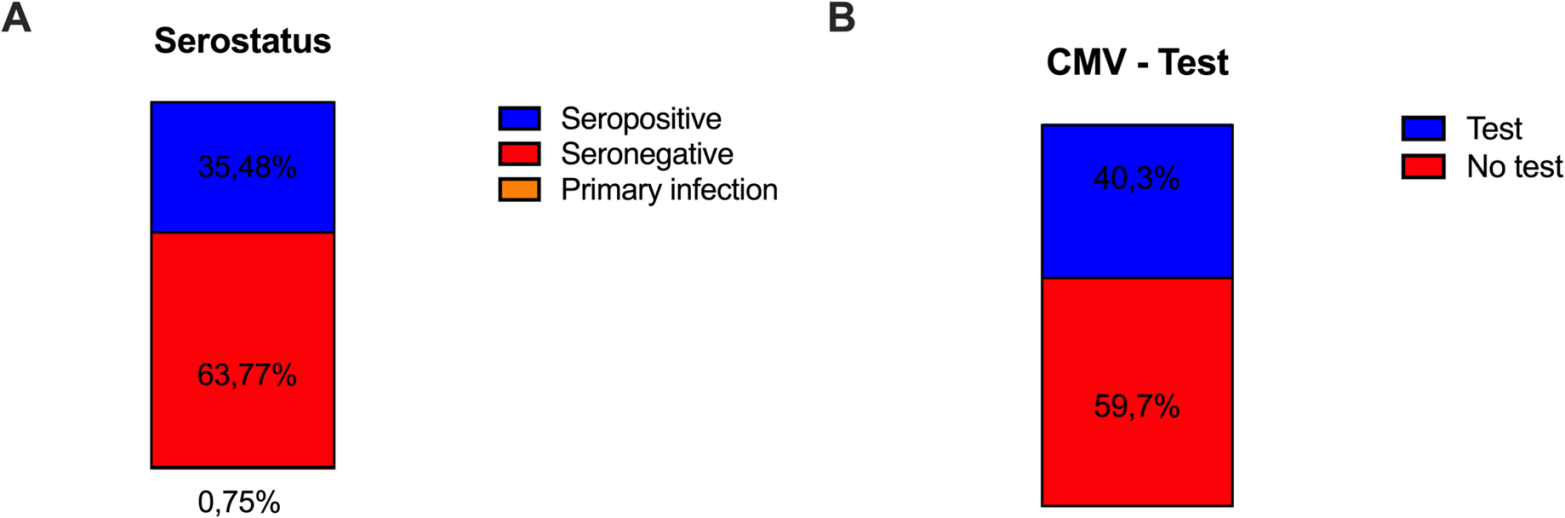
Overview of **A** Serostatus, **B** CMV testing

CMV test was performed in 403 patients (40.3%). Among these, 143 (35.5%) were seropositive, 257 (63.8%) showed no IgG and IgM antibodies and three (0.75%) had a primary infection (Fig. 1A, B).

A subgroup of 15 patients was identified, who underwent a CMV screening test in a previous pregnancy, i.e. during their second pregnancy, of which 13 were secundipara, one was para-3 and one para-4. 11 of 15 women were seropositive, while four were seronegative.

### Primary infections during pregnancy

In this study, three primary CMV infections were recorded in patients, aged between 29–38 years. Patient A, a secundipara, had a periconceptional CMV infection and underwent a CMV screening at 12 weeks. Patient B, who was a primipara, was tested at 11 weeks of gestation and had a primary infection in the first trimester, while Patient C, a secundipara, was also screened at 11 weeks and had a periconceptional CMV infection. A treatment with CMV-HIG (hyperimmune globulin), in a dosage of 200 IU/kg BW (body weight) i.v. (intravenous), was administered in all three patients biweekly, starting at 11 to 14 until 19 to 21 weeks. The detailed fetal scan, performed in the second trimester, showed no abnormal findings at any of the patients. An amniocentesis was suggested to the three pregnant women in the second trimester in order to investigate whether a vertical transmission had taken place. Only patient A consented to the procedure, showing no evidence of CMV DNA. After delivery, a qPCR CMV test was performed using blood sample from the umbilical cord as well as urine sample from the newborns within the first 48 h after birth. All three newborns showed negative results for a CMV infection.

### Premature birth rate

In our set of data, 108 (10.8%) premature births were recorded. In 70 women (64.8%) no CMV screening test was conducted, while 38 (35.1%) were tested and 31 of them were seronegative.

In this subgroup, we analyzed whether the CMV serostatus can affect the risk of premature birth. A statistically significant difference was found between the subgroups mentioned above: less CMV seropositive women had a preterm birth compared to seronegative ones (*p* = 0.02, OR = 0.375, 95% CI = 0.154–0.865) (Table 1).

**Table 1:** Premature birth rate in relation to serostatus: a statistically significant difference was found between seropositive and seronegative women in the premature birth group; more seronegative women had a preterm birth compared to those, who were seropositive

Data analyzed	Preterm birth	Full-term birth	Total
Seropositive	7	136	143
Seronegative	31	226	257
Total	38	362	400

### Gestational age at delivery

The differences in the gestational age at delivery between seropositive and seronegative patients as well as those with a primary CMV infection, were compared. No significant difference in gestational age between the groups was found (*p* = 0.27, Median: 40 vs. 40) (Table S1, S2).

### Age of pregnant women undergoing a CMV test

**Fig. 2:**
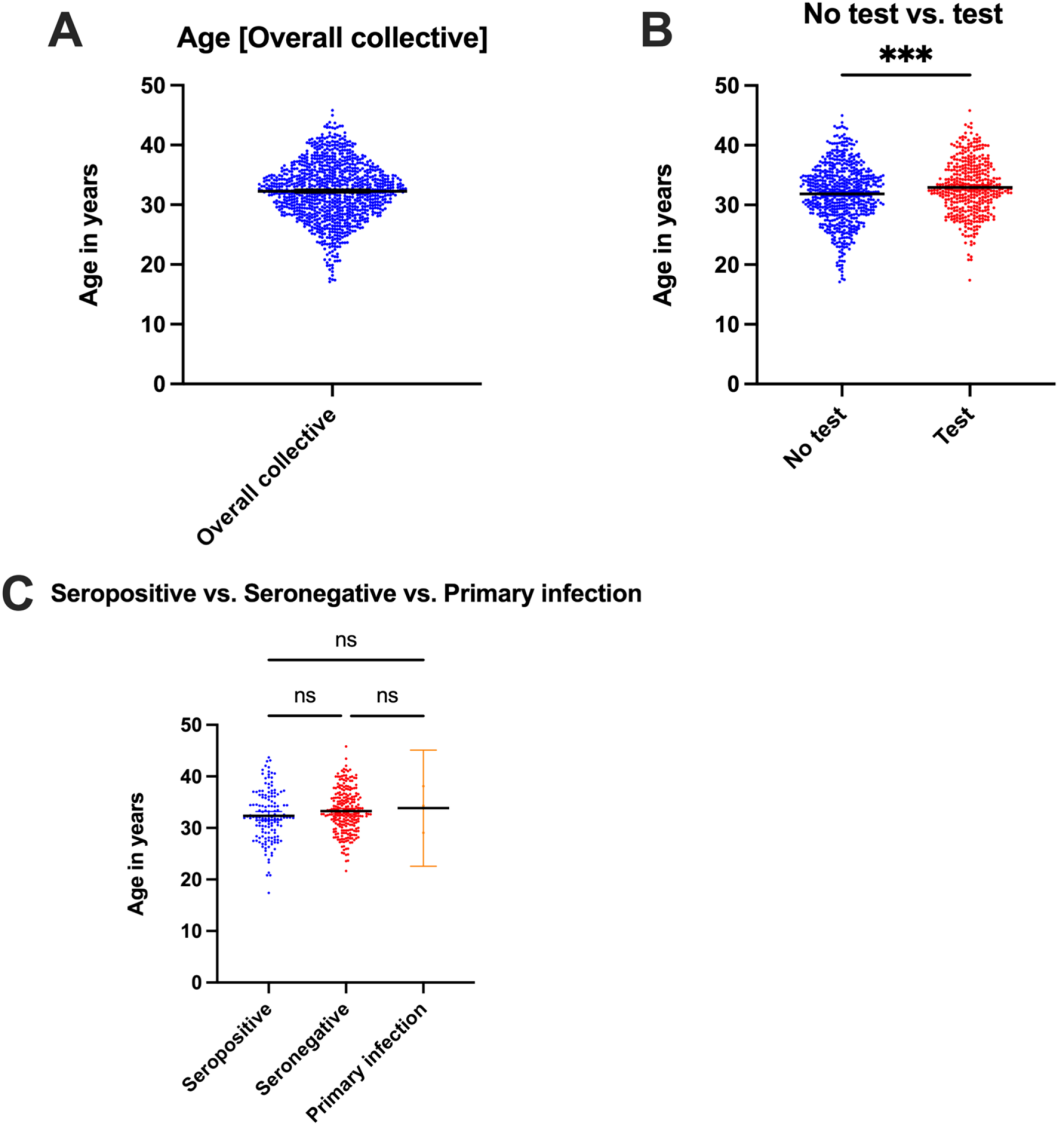
CMV testing related to age and serostatus. **A** Overall collective. **B** Women tested were on average older than the patients without a test. **C** No significant difference was revealed between the three subgroups of patients receiving test

The statistical analysis indicated that women undergoing a CMV screening test were on average older than the patients without (Mean: 32.94 vs. 31.85 years, *p* = 0.0004) (Fig. 2B) (Table S3, S4).

Comparing the three subgroups seropositive vs. seronegative vs. primary infection, no significant difference in age was revealed (Mean: 32.35 vs. 33.26 vs. 33.84 years, *p* = 0.29) (Fig. 2C) (Table S5, S6).

## Discussion

In this study, we assessed for the first time in Upper Palatinate, Bavaria the frequency of CMV testing during pregnancy as an individual healthcare service. We evaluated the data of 1000 pregnant women that were treated and gave birth in our clinic, which is a highly representative local German cohort.

According to our results, most of the patients did not receive a CMV screening test (59.7%) during their pregnancy. Most of the tested patients, were CMV seronegative (63.8%). The prevalence of a primary CMV-infection (0.75%) in our cohort turned out to be significantly low.

The data regarding the CMV seroprevalence is heterogenic. In industrialized countries is about 42.3%−68.3% among adults [17], with great regional differences existing [18]. Available data regarding the CMV seroprevalence among pregnant women in Germany is also rather limited. Enders et al. described a seroprevalence among pregnant women of 42.3% [19]. Our results revealed an overall seroprevalence of 36.2%.

Concerning the likelihood of a vertical transmission during primary compared to non-primary infection, most authors report a higher transmission rate in case of a primary infection. The transmission rate varies internationally enormously [20–22]. The prevalence of cCMV (congenital cytomegalovirus infection) at birth increases with increasing maternal CMV prevalence. A possible explanation is that with a high seroprevalence in the population, the risk of CMV reactivation/reinfection is greater, and this risk exceeds the protective effect of maternal CMV seropositivity regarding transplacentar transmission [4, 23, 24]. For example, the cCMV prevalence at birth in countries like Brazil is 1.1%, where the seroprevalence in the population is about 96%. Similarly high prevalence of cCMV can be found in Africa (0.9–1.4%) [25], China (1.8%) [26] and India (2.1%) [27], where the CMV seroprevalence is almost 100%. In contrary, the prevalence of cCMV fluctuates in Europe and the USA between 0.18% to 0.48% with a much lower CMV seroprevalence of about 30–40% [28].

Hygiene measures, especially for pregnant women with high occupational or familial CMV exposure, are the most important components of primary prevention of cCMV infection. Several studies have shown that by advising pregnant women about the significance of cCMV infection and the possible hygiene measures, especially during the first trimester, the risk of infection can be significantly minimized, thus contributing immensely to the prevention of infection [29–31]. In our patient population, it is unclear whether the patients were informed about the primary prevention measures by their gynecologists during maternal care. However, the study took place during the COVID-19 pandemic, so it can be assumed that during this time the entire population, including our study participants, were extensively informed about hygiene measures (keeping distance, hygiene, everyday masks) and followed them, which could influence our results and explain the lower CMV seroprevalence values (36.2%) compared to the literature data regarding pregnant women in Germany.

In case of a CMV infection during pregnancy measures need to be taken to avoid vertical transmission. In our study three patients with a primary CMV infection were detected. Since the study took place in 2021, where HIG administration was considered to be still state of the art, HIG was administered “off-label" biweekly starting before or at 14 weeks in a dose of 200 IU/ kg BW, based on the studies of Kagan et al. [32, 33]. Umbilical cord blood samples and urine samples of the three newborns were tested postnatally by PCR for CMV DNA with negative results, proving that no vertical transmission had taken place.

CMV-HIG administration is an option of treatment, which has been controversially discussed. According to the prospective, randomized trial of Revello et al., HIG treatment cannot reduce the risk of transmission (30% HIG group vs. 44% placebo group; *p* = 0.13) [34]. On the other hand, Kagan et al., showed that HIG treatment was effective and that the risk of materno-fetal transmission could be significantly reduced (7.5% vs. 35.2%; *p* < 0.0001).

According to a RCT (randomized controlled trial) in 2020 and following quasi-randomized trials, through administration of oral 8 g/day valacyclovir, a reduction of 70% in the vertical transmission rate in case of a maternal primary infection acquired periconceptionally or during the first trimester, can be achieved, if initiated as soon as possible [16]. Therefore, since last year the standard treatment in our center in case of a primary infection is an antiviral therapy with an oral administration of 8 g/day of valacyclovir.

Lereuz-Ville et al. have evaluated the efficacy of antiviral treatment with valacyclovir and demonstrated that high-dosage valacyclovir is effective in improving the outcome of moderately symptomatic fetuses [16]. An amniocentesis was performed at one of our patients with a primary infection at 21 weeks and a PCR test was carried out to detect CMV DNA, which in the case of our patient was negative, so a further treatment with valacyclovir was not necessary. The other two patients rejected an amniocentesis, but the detailed ultrasound scan performed showed no signs of CMV infection and the newborns turned out to be negative.

Moreover, 108 premature births were recorded. Most of these women did not undergo a CMV screening (64.8%, *n* = 70). Considering the ones that were tested for CMV during pregnancy (35.1%, *n* = 38), 31 (12.07%) were seronegative. Looking at the total patient population (*n* = 1000), no significant difference regarding the gestational age at birth between the seropositive and seronegative patients could be observed (*p* = 0.27).

A possible explanation for the latter results is that seronegative women are at high risk of a primary CMV infection. CMV can influence the development and function of placenta through various immunological pathways leading to an increased risk of intrauterine growth restriction, premature birth and still birth [35]. Premature delivery is associated with lower socioeconomic status [36–38]. There may be a co-incidence of several risk factors for preterm birth and a higher probability to miss CMV testing. Since CMV testing in Germany is optional and cost-intensive for the individual, this aspect may mainly contribute to our findings that women with preterm birth are at higher risk not to be tested.

Our study showed that, pregnant women with a CMV test were significantly older than those without, with a mean age of 32.94 years. In this case, it can be assumed, that the older the pregnant woman is, the more likely it is, that the pregnancy was planned, and that more individual health services are used. In addition, older patients may already have small children who represent a CMV reservoir. Accordingly, a CMV test was carried out on these patients with an increased risk of CMV exposure as part of primary prevention. Among the subgroups of patients, who were tested (seropositive, seronegative, primary infection), no significant age difference was observed.

Although CMV is the most common cause of viral congenital infections and can strongly impact the health of affected newborns, in Germany there is no antenatal screening program. A favorable testing procedure would suggest that if a pregnant woman is seropositive during the first trimester, no further measurements need to be taken. In case of seronegativity, the patients should be advised to repeat the test in the second and third trimester, so that a seroconversion can be detected. In the case of a diagnosed primary infection during pregnancy, an antiviral treatment with oral administration of 8 g/day of valacyclovir should be recommended, followed by a CMV PCR test using amniotic fluid sample obtained by amniocentesis at about 20 weeks, to assess the need for further treatment with valacyclovir until the end of a pregnancy in case of a vertical transmission. We state that this all should be offered free in our opinion for the pregnant women in Germany.

Data collected from other European countries, e.g. Austria, Italy, Greece, Belgium, and certain regions of France, where CMV testing is part of routine screening, have contributed to better research and understanding of the pathophysiology of congenital CMV, CMV diagnostics, and the importance of hygiene as a preventive measure [39–42].

To date, despite extensive research, neither a vaccine nor an approved therapy is available. However, the administration of an antiviral therapy (“off-label”) has shown good preventive and therapeutic outcomes. Therefore, early diagnosis and hygiene measures remain the most important means for preventing maternal seroconversion and consequently, a potential vertical infection of the fetus.

## Conclusion

Although CMV can severely impair the health of affected newborns in case of congenital infection, CMV diagnostics during pregnancy is in Germany still an individualized health service. Under current conditions, more than half of pregnant women in Germany do not undergo CMV testing. Unfortunately, there is currently no vaccination available to prevent vertical transmission, which is why early diagnosis in the first trimester as well as hygiene measures, are the most important means of preventing seroconversion of the mother and possible infection of the fetus.

Our results strongly indicate the importance of a general and free of charge CMV testing during pregnancy in Germany.

## Supplementary Information

Below is the link to the electronic supplementary material.

Supplementary file1 (XLSX 59 KB)

Supplementary file2 (XLSX 58 KB)

Supplementary file3 (XLSX 58 KB)

Supplementary file4 (XLSX 59 KB)

Supplementary file5 (XLSX 58 KB)

Supplementary file6 (XLSX 59 KB)

## Data Availability

The data that support this study are not openly available due to reasons of sensitivity, since a participant consent to share the data publicly is not available.
